# Using New Science to reduce bias of Old Science: AI‐driven Customization of Culturally Biased Tests

**DOI:** 10.1002/alz.091267

**Published:** 2025-01-09

**Authors:** Sherral A. Devine, Melissa Lee, Ashita S. Gurnani, Preeti Sunderaraman, Lampros Kourtis, Abhishek Pratap, Spencer Low, Kristi Ho, Katherine A. Gifford, Rhoda Au

**Affiliations:** ^1^ Department of Anatomy & Neurobiology, Boston University Chobanian & Avedisian School of Medicine, Boston, MA USA; ^2^ Framingham Heart Study, Boston University Chobanian & Avedisian School of Medicine, Boston, MA USA; ^3^ Alzheimer's Drug Discovery Foundation, New York, NY USA; ^4^ Department of Neurology, Boston University Chobanian & Avedisian School of Medicine, Boston, MA USA; ^5^ Boston University Alzheimer's Disease Research Center, Boston, MA USA; ^6^ Boston University Chobanian & Avedisian School of Medicine, Boston, MA USA; ^7^ Gates Ventures, Seattle, WA USA; ^8^ Clinical and Translational Science Institute, Tufts University, Boston, MA USA; ^9^ Boehringer Ingelheim, Ingelheim am Rhein Germany; ^10^ Boston University, Boston, MA USA; ^11^ Davos Alzheimer's Collaborative, Philadelphia, PA USA; ^12^ Department of Public Health, School of Public Health, Boston University, Boston, MA USA; ^13^ Department of Neurology, Vanderbilt University Medical Center, Nashville, TN USA; ^14^ Department of Medicine (Biomedical Genetics), Boston University Chobanian & Avedisian School of Medicine, Boston, MA USA; ^15^ Department of Epidemiology, Boston University School of Public Health, Boston, MA USA; ^16^ Boston University Chobanian & Avedisian School of Medicine and School of Public Health, Boston, MA USA

## Abstract

**Background:**

Widely used neuropsychological test instruments are notoriously biased across the demographics of age, sex/gender, education, language and culture. This includes verbal memory tests that elicit speech such as the paragraph recall or list‐learning memory tests. Language tests are similarly biased, including the Boston Diagnostic Aphasia Examination Cookie Theft Test (CTT) that has been used to elicit both written and spoken responses for decades. Biased tests generate biased results, and this problem is exacerbated by the widespread practice of repurposing tests generated for those in largely high income, Westernized countries for use in other countries that are demographically and culturally distinct. We tested the use of large language model (LLM) tools to automatically generate verbal test stimuli that were more locally relevant.

**Methods:**

Starting with the CTT, that involves describing a picture that contains 14 core content units, we used ChatGPT to aid in generating customized pictures with the same number of core content units that were more universally relevant, (e.g., open park area, beach, etc.) by suggesting parameters and integrating resulting features into new pictures. We also tested capabilities to generate pictorial scenes that are more representative of regional settings, such as the Asia Pacific or Middle East. We are testing LLM capacity to generate similarly regionally customized list learning stimuli that maintain the original list learning test word frequency and difficulty across languages.

**Results:**

Using an iterative approach, we were able to use automated artificial intelligence (AI)‐driven tools to rapidly generate regionally relevant test stimuli that retained the original test characteristics to preserve generation of comparable results. Descriptive comparisons found that aided by automated AI tool, we could generate new neuropsychological test stimuli in a matter of minutes compared to months long manual methods.

**Conclusion:**

The rapid emergence of AI has generated significant concerns about its appropriate use for health and health care applications. Given that AI is here to stay, we are testing the capacity of this technological advance to address long‐standing, persistent problems in AD research and usher in a new era of precision brain health that has global relevance in a rapidly aging world population.

